# Relationship between Insulin Resistance Risk Scales and Non-Alcoholic Fatty Liver Disease and Liver Fibrosis Scales in 219,477 Spanish Workers

**DOI:** 10.3390/metabo12111093

**Published:** 2022-11-10

**Authors:** José Ignacio Ramírez-Manent, Emilio Martínez-Almoyna, Carlos López, Carla Busquets-Cortés, Hilda González San Miguel, Ángel Arturo López-González

**Affiliations:** 1 ADEMA-SALUD Group of IUNICS, University of Balearic Islands, 07009 Palma, Spain; 2 Balearic Island Health Service, 07003 Palma, Spain; 3 Department of Medicine, University of the Balearic Islands, 07120 Palma, Spain

**Keywords:** liver fibrosis, non-alcoholic fatty liver disease, type 2 diabetes, insulin resistance, scale risk

## Abstract

Insulin resistance (IR) has been identified as a key factor in the appearance of non-alcoholic fatty liver disease (NAFLD) as it is related through a complex molecular biochemical and immunological mechanism. Our aim was to evaluate the relationship between different scales that estimate the risk of IR and scales that determine the risk of NAFLD. This descriptive and cross-sectional study was performed in 219,477 Spanish workers from different sectors and Spanish regions. The prevalence of high values of all the IR scales increases as the values of the NAFLD and liver fibrosis scales increase. In the multivariate analysis, the risk of presenting high values in the IR scales increases greatly as the values of the NAFLD and hepatic fibrosis scales increase, with particularly high OR values when using the Metabolic Score for Insulin Resistance (METS-IR) scale (LAP high OR 42.20 (95% CI (39.10–45.56) and FLI high OR 32.35 (95% CI 31.10–33.61)). We can conclude that there is a direct relationship between the values of the IR scales and the NAFLD and liver fibrosis scales in our population.

## 1. Introduction

Insulin is a pancreatic hormone that is released by increased blood glucose levels after food intake. Insulin facilitates an increase in glucose transport to liver, muscle, and adipose tissue where phosphorylation occurs and it is transformed into glucose-6-phosphate, increasing glycogen formation and protein synthesis. In this way, it decreases the high levels of glucose in the blood and promotes its use by the tissues [1].

Insulin resistance (IR) is defined as a situation of diminished response to physiological levels by the tissues on which this hormone acts, thus reducing their ability to assimilate and use blood glucose [1].

In adipose tissue, there is an increase in the lipolysis that was prevented by insulin, with a rise in circulating free fatty acids (FFA) that block the antilipolytic action of insulin [2]. In muscle, FFAs inhibit protein kinase activation which decreases glucose uptake. At the hepatic level, FFAs stimulate the activation of protein kinase, increasing glycogenesis and lipogenesis. To maintain a state of euglycemia, insulin levels increase, leading to chronic hyperinsulinemia, which turns into deficiency of pancreatic beta cells that trigger type 2 diabetes (T2D) [3]. IR promotes the development of non-alcoholic fatty liver disease (NAFLD) [3], gestational diabetes [4], metabolic syndrome [5], obesity [6], hypertriglyceridemia [7] and arterial hypertension [8].

The diagnosis of IR is performed by checking insulin in blood levels, which is an aggressive technique. There are scales that, even with no exact diagnosis, contribute to assess the risk of presenting IR [9].

NAFLD is a pathological entity characterised by the accumulation of fat in the liver in people who do not consume high amounts of alcohol, and which is generally related to being overweight. Some authors have assessed the role of IR in the appearance of NAFLD [10]. On the other hand, liver fibrosis is the massive accumulation of extracellular matrix proteins, such as collagen, that arises in most types of chronic liver diseases, including NAFLD, and can precede cirrhosis or liver failure [11]. IR, T2D, NAFLD and liver fibrosis are related through a complex molecular biochemical and immunological mechanism. In fact, NAFLD is strongly associated with IR such that the occurrence of NAFLD is five-fold higher in subjects with T2D compared with those without [12]. NAFLD currently affects 25% of adults and is becoming more frequent in children, which is a public health problem worldwide [13]. The gold standard for the diagnosis of NAFLD is liver biopsy, which is an invasive, expensive procedure with many serious side effects. Therefore, developing non-invasive methods that can detect early NAFLD in patients with T2D seems to be a priority. Attempts have been made to diagnose NAFLD and liver fibrosis using clinical biomarkers and scoring scales that can estimate fatty changes in the liver. These indices for the diagnosis of NAFLD include the fatty liver index (FLI), the hepatic steatosis index (HSI), Zhejian University index (ZJU), the fatty liver disease index (FLD), triglyceride–glucose index (TyG index), Framingham steatosis index (FSI), the lipid accumulation product (LAP), and the BARD scoring. These indices require the measurement of biochemical and anthropometrical parameters, including concentrations of triglycerides (TGs), fasting plasma glucose (FPG), γ-glutamyl transpeptidase (GGT), aspartate aminotransferase (AST), alanine transaminase (ALT), HDL-c (high-density lipoprotein cholesterol), LDL-c (low-density lipoprotein cholesterol), insulin, body mass index (BMI), waist circumference (WC), gender, and the presence or absence of T2D or MetS, among others. Due to the aggressive techniques for diagnosis and their high cost, we consider it interesting to carry out a study on the risk scales of these processes, since they can be just as effective, less aggressive, more efficient, and easier to apply by the clinician.

Our aim is to evaluate the relationship between different scales that estimate the risk of IR and scales that determine the risk of NAFLD in a group of Spanish workers.

## 2. Materials and Methods

### 2.1. Participants

**Figure 1:**
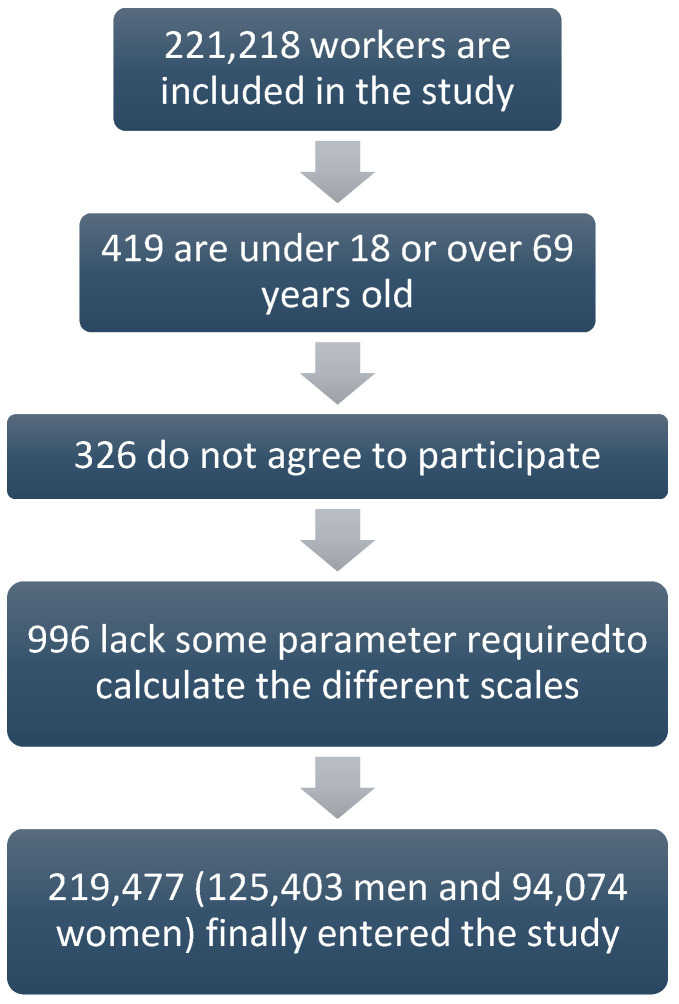
Flow chart of study participants.

The present descriptive and cross-sectional study was performed in 219,477 workers from different autonomous communities of Spain (Balearic Islands, Canary Islands, Andalusia Valencian Community, Madrid, Catalonia, Castilla y León, Castilla La Mancha, and Basque Country) belonging mainly to labour sectors of public administration, health, construction, and commerce. The participants were selected from labour medical examinations between the months of January 2017 and December 2019 of the different companies that participated in the study. Participants were recruited when they met the following inclusion criteria: age from 18 to 69 years, belonging to one of the companies included in the study and not being in a situation of temporary disability, providing written consent to participate in the study and giving permission for using their data for epidemiological purposes. Figure 1 shows the flowchart of the participants in the study.

### 2.2. Measurements and Data Collection

Standard health examinations, anthropometric measurements, and metabolic tests were performed at baseline to all participants. Anthropometric (height and weight), clinical and analytical measurements were performed by personnel from the different occupational health units participating in the study, after standardisation of the measurement techniques. Weight (in kg) and height (in cm) were determined with a SECA 700 scale with a SECA 220 telescopic measuring rod attached. Waist circumference (WC) was measured with a SECA tape measure with the person in a standing position, feet together and trunk erect, with the abdomen relaxed. The tape was placed parallel to the ground at the level of the last floating rib. The waist/height index was obtained by dividing the WC in cm by the height in cm. The cut-off point was 0.50 [14]. BMI was calculated by dividing weight by height in square meters.

Blood pressure was determined with the person seated and after 10 min of rest. A calibrated OMRON M3 automatic sphygmomanometer was used. Three determinations were made with one-minute intervals and the mean of the three was obtained. Blood was obtained after 12 h of fasting. The samples were sent to reference laboratories and processed within 2–3 days. Automated enzymatic methods were used to determine glucose, total cholesterol, and TG. HDL-c was determined by precipitation with dextran sulfate-MgCl_2_. LDL-c was calculated by the Friedewald formula (providing that TG was less than 400 mg/dL). The values of all these parameters are expressed in mg/dL.

Friedewald formula:

LDL = total cholesterol − HDL − triglycerides/5

The following insulin resistance scales were determined:

- TyG index [15]: triglyceride–glucose index.

TyG index = LN (TG (mg/dL) × glycemia [mg/dL]/2)

- Metabolic score-insulin resistance (METS-IR) [16]

METS-IR was calculated from the following formula:

Ln (2 × glycemia + triglycerides) × BMI/Ln (HDL)

Glycemia, triglycerides and HDL are expressed in mg/dL.

- Triglycerides/HDL-c

The NAFLD and liver fibrosis risks were determined by:

- Fatty liver index (FLI) [17]

FLI was calculated with the following formula:

(e0.953 × loge (triglycerides) + 0.139 × BMI + 0.718 × loge (GGT) + 0.053 × waist − 15.745)/(1 + e0.953 × loge (triglycerides) + 0.139 × BMI + 0.718 × loge (GGT) + 0.053 × waist − 15.745) × 100

FLI cut-off points were set as: low risk: <30 points; moderate risk: 30–59 points; and high risk: >60 points.

- Hepatic steatosis index (HSI) [18]

HSI was calculated with the following formula:

8 × AST/ALT + BMI + 2 if diabetes is present, + 2 if female

The cut-off points were established as follows: low risk: <30 points; moderate risk: 30–35.9 points; and high risk: >36 points.

- Zhejian University index (ZJU index) [19]

ZJU index was calculated according to the following formula:

BMI + glycemia (mmol L) + triglycerides (mmol L) + 3 AST/ALT + 2 if female

The cut-off points were established as follows: low risk: <32 points; moderate risk: 32–37.9 points; and high risk: >38 points.

- Fatty liver disease index (FLD) [20]

FLD was obtained from the following formula:

BMI + triglycerides + 3 × (AST/ALT) + 2 × hyperglycaemia (presence = 1; absence = 0).

The cut-off points were established as: low risk: <28 points; moderate risk: 28–36.9 points; and high risk: >37 points.

- Framingham steatosis index (FSI) [21]

FSI was calculated by applying the following formula:

FSI = −7.981 + 0.011 × age (years) − 0.146 × sex (female = 1; male = 0) + 0.173 × BMI (kg/m2) + 0.007 × triglycerides (mg/dL) + 0.593 × hypertension (yes = 1; no = 0) + 0.789 × diabetes (yes = 1; no = 0) + 1.1 × ALT/AST ratio ≥ 1.33 (yes = 1; no = 0)

- Lipid accumulation product (LAP) [22]

LAP was calculated by the formula.

- In men:

(WC (cm) − 65) × (TG concentration (mMol))

- In women:

(WC (cm) − 58) × (TG concentration (mMol))

Values above 42.7 indicate high risk of NAFLD.

- BARD scoring [23]

This is a scale that assesses the risk of liver fibrosis in NAFLD patients.

The presence of a BMI over 28 is punctuated with 1 point, a GOT/GPT ratio over 0.8 is punctuated with 2 points and the presence of diabetes mellitus is also punctuated with 2 points. Values between 2–4 points indicate high risk of liver fibrosis.

A smoker was considered a person who had smoked at least one cigarette/day (or its equivalent in other types of consumption) in the last 30 days, or who had quit smoking less than 12 months ago. A person who had not smoked in the last year or had never smoked was considered a non-smoker. Social class was determined from the National Classification of Occupations 2011 (CNO-11) according to the proposal of the social determinants group of the Spanish Society of Epidemiology [18]. Three categories were established: Class I: directors/managers, university professionals, athletes, and artists; Class II: intermediate occupations and skilled self-employed workers; Class III: unskilled workers. Participants were considered overweight if their body mass index (BMI, kg/m^2^) was 25 or greater, and obese if their BMI was 30 or greater. Obesity was further separated into 3 classes according to the increased health risks linked with increasing BMI levels: class I (BMI 30–34.9); class II (BMI 35–39.9); and class III (BMI ≥ 40).

### 2.3. Statistical Analysis

A descriptive analysis of the categorical variables was performed, calculating the frequency and distribution of responses for each of them. For quantitative variables, the mean and standard deviation were calculated by following a normal distribution. The bivariate association analysis was performed using the chi2 test (with correction of Fisher’s exact statistic when conditions required it) and Student’s *t*-test for independent samples (for comparison of means). Multivariate techniques were used to establish the variables associated with the most significant risk factors. For the multivariate analysis, logistic regression was used, with calculation of the odds ratio and the Hosmer–Lemeshow goodness-of-fit test. ROC (Receiver Operating Characteristic) curves were determined by establishing the areas under the curve of the different insulin resistance risk scales in relation to the NAFLD and liver fibrosis scales. The statistical analysis was performed with the Statistical Package for the Social Sciences (SPSS) version 28.0 software (IBM Company, New York, NY, USA) for Windows, with an accepted statistical significance level of 0.05.

### 2.4. Ethical Considerations and/or Aspects

The research team was always committed to following the ethical principles of health sciences research established at the national and international level (Declaration of Helsinki), paying special attention to the anonymity of the participants and the confidentiality of the data collected. We requested the approval of the Balearic Islands Research and Ethics Committee (CEI-IB), which was obtained with the following indicator IB 4383/20. Participation in the study was voluntary so that the participants consented, orally and written, to participate in the study after having received sufficient information about the nature of the study. For this purpose, they were given an informed consent form, as well as an information sheet, explaining the aim of the study.

The data collected for the study were identified by a code and only the person responsible for the study can relate these data to the participants. The identity of the participants will not be disclosed in any report of this study. The investigators will not disseminate any information that could identify them. In any case, the research team undertakes to strictly comply with Organic Law 3/2018, of December 5, on the protection of personal data and guarantee of digital rights, guaranteeing the participant in this study that he/she may exercise his/her rights of access, rectification, cancellation, and opposition of the data collected.

## 3. Results

Table 1 shows the baseline anthropometric and clinical characteristics of the participants. A total of 125,403 men (57.14%) and 94,074 women (42.86%) were included in the analyses. The average age of the sample was 40.5 ± 10.5 years, with the majority group being between 30 and 49 years. The anthropometric, clinical, and analytical values were higher in males. Most workers (75.5%) belonged to social class III. A total of 33.3% of women and 32.5% of men were smokers. The percentage of patients with obesity I was 14.2%, 4.2% of the total sample had obesity II, and 1.5% of the population was classified into the obesity III category.

Table 2 shows how the values of the different NAFLD and liver fibrosis risk scales increase as the values of the insulin resistance risk scales increase. These results were obtained in both sexes.

Table 3 shows that the prevalence of high-risk values on the NAFLD and liver fibrosis scales is higher in people who present high values on the insulin resistance risk scales in both sexes. In all cases the differences are statistically significant (*p* < 0.001).

In the multivariate analysis (Table 4) we observed that, in all cases, high values on the risk scales for insulin resistance increased the risk of presenting high values on the risk scales for NAFLD and liver fibrosis, with statistically significant differences (*p* < 0.0001) in all cases. The highest OR values are found with high METS-IR.

**Figure 2:**
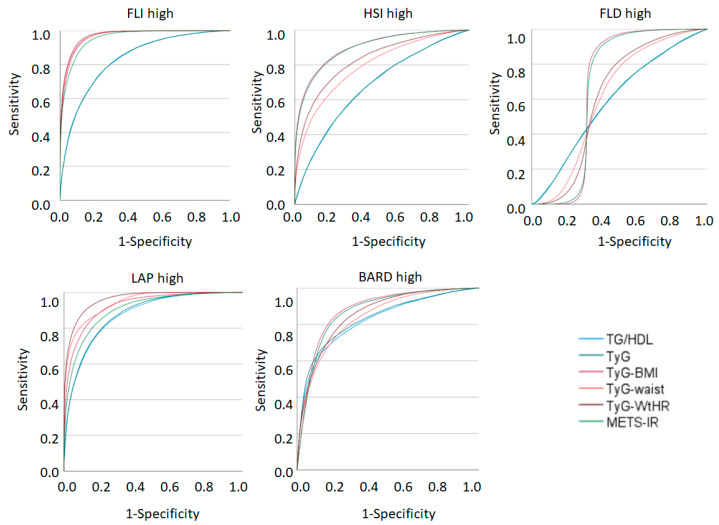
Areas under the curve of insulin resistance risk scales to predict the presence of high values of the NAFLD and liver fibrosis risk scales. TG: triglycerides. HDL-c: high-density lipoprotein cholesterol. TyG: triglyceride–glucose. METS-IR: metabolic score for insulin resistance. BMI: body mass index. WtHR: waist-to-height ratio. FLI: fatty liver index. HSI: hepatic steatosis index. ZJU: Zhejiang University. FLD: fatty liver disease index. FSI: Framingham steatosis index. LAP: lipid accumulation product.

Figure 2 and Table 5 show the areas under the curve of the insulin resistance risk scales with their 95% confidence intervals to predict the presence of high values of the NAFLD risk scales and hepatic fibrosis. The largest areas under the curve are found with high FLI and high LAP, while the lowest values are presented by high NAFLD.

## 4. Discussion

The main feature of NAFLD is the accumulation of fat in the liver that is not associated with alcohol consumption but generally related to being overweight. The role of IR in the appearance of NAFLD has been assessed. Thus, IR, T2D, and NAFLD appear to be connected, not only through a complex molecular biochemical mechanism, but also through immunological mechanisms. The prevalence of NAFLD is over 25% in the adult population and it is becoming more frequent in children as well. Therefore, a public health problem with epidemic proportions is on the horizon for society. The gold standard for the diagnosis of NAFLD is liver biopsy, which is an invasive, high-priced, and painful procedure. Additionally, performing liver biopsies in all patients is not realistic in clinical practices. The reduction of side-effects and mortality rates are a main concern in healthcare institutions. Thus, developing non-invasive methods that can detect early NAFLD in patients with T2D seems to be a priority. An appropriate alternative is the development of risk scales, which permit monitoring of the clinical evolution and improve clinical outcomes in their patients The medical community has noticed the necessity to generate and develop tools such as algorithms, scales and scores to target early patients at risk for different pathological conditions. Therefore, different scales have been developed in search of an optimal scale for the diagnosis of NAFLD. The first to be designed were LAP and FLI (2005 and 2006), later HSI (2010), FLD (2013), BARD score (2016), and recently the ZJU index (2019) and FSI (2021). Although none of them is especially recommended by any scientific society, they are widely used in epidemiological studies due to their ease of application and low cost. Of all of them, the most extensively used is FLI.

These tools are based on easy data collection and simple clinical interpretations, allowing the clinical personnel to make an objective and early diagnosis of the state of the patients [24]. In our work, we selected those formulas most generally used and for which we had all the data for their calculation. In our study, we worked with these scales to try to obtain the most effective and efficient scale that can be applied in medical clinics.

In the analyses carried out in our study, a good relationship was observed between the values of the NAFLD and liver fibrosis risk scales with the values of the insulin resistance risk scales. The good results obtained in the AUC indicate that all the fatty liver risk formulas are good predictors except for ‘FLD High’, which presented an AUC ranging between 0.5 and 0.67 for the different scales of insulin resistance, which indicates poor test results. In our opinion, the AUC should be similar because they share most of the parameters for their calculation, so we did not find an explanation for lower FLD-high values. 

In the rest of the formulas, the AUC obtained for FLI high and LAP high standout with values between 0.83 and 0.97. In these results, the AUC obtained between FLI high and TyG–WtHR stands out with a result of 0.972, that is, an excellent test. This may be due to the fact that they are the only scales that include the waist circumference in their calculation.

Many studies have shown a close relationship between insulin resistance and NAFLD and liver fibrosis, although these have generally been diagnosed through objective tests and not through risk scales [3,25]. In our work, we found a close relationship between insulin resistance scales and formulas for detecting NAFLD using non-invasive procedures. 

A study conducted in the Romanian population by Popa et al. [26] showed a close relationship between insulin resistance and FLI values. This is concordant with the results obtained in our study, where the best AUC results were obtained for FLI high. Petta et al. found a good association between insulin resistance and NAFLD assessed by HSI [27]. They also found a good relationship with the risk of liver fibrosis, although in this case it was assessed with FIB-4 and not with BARD scoring as we did in our work. A Spanish study by Bullón-Vella et al. derived from the PREDIMED [28] study found a good relationship between IR determined by the TyG index and NAFLD determined with FLI and HSI. This coincides with the results of our study where the AUC for the TyG index and FLI high presented results of 0.83. The study by Ji et al. in a Chinese population [29] assessed the usefulness of the ZJU index to determine the presence of insulin resistance and concluded that the ZJU index is a useful indicator to recognize IR in the general Chinese population. A Korean study by Chun et al. [30] related hepatic fibrosis assessed by BARD scoring and insulin resistance, finding a good association. This is consistent with our work in that the AUC for BARD high was greater than 0.8 on all the used IR risk scales.

All the evidence above justifies a greater use of the scales in patients suffering from NAFLD, since they avoid aggressive techniques such as liver biopsy, and do not make imaging techniques necessary. None of the NAFLD scales use expensive variables. In epidemiological studies with large sample sizes such as ours, the most difficult parameter to obtain due to its high price is insulinemia; however, both the TyG index and triglycerides/HDL or METS-IR can replace HOMA (homeostatic model assessment).

The most positive aspect is the ease of application of all the scales, with very low cost and high correlation.

### Strengths and Limitations

As strong points of the study, we highlight the large sample size (more than 200,000 people) and the large number of NAFLD, liver fibrosis and insulin resistance risk scales used. As a main limitation, we did not perform diagnostic techniques for NAFLD, liver fibrosis or IR other than risk scales.

## 5. Conclusions

Considering the results obtained in our study, we can conclude that in this Spanish working population there is a direct relationship between the values of the different insulin resistance scales analysed and the values of the NAFLD and liver fibrosis scales used. From the economic point of view, all the scales used are cheap and have a good correlation.

## Figures and Tables

**Table 1:** Characteristics of the population.

	Men *n* = 125.403	Women *n* = 94.074	
	Mean (SD)	Mean (SD)	*p*-Value
**Age**	41.8 (10.5)	39.9 (10.5)	<0.0001
**Height**	175.2 (6.8)	162.3 (6.3)	<0.0001
**Weight**	82.6 (15.0)	68.0 (14.7)	<0.0001
**SBP**	126.1 (15.6)	115.4 (15.5)	<0.0001
**DBP**	77.3 (11.1)	72.3 (10.5)	<0.0001
**Cholesterol**	195.6 (37.9)	192.1 (35.5)	<0.001
**HDL-c**	52.1 (9.8)	57.2 (10.3)	<0.0001
**LDL-c**	118.4 (35.1)	116.3 (33.5)	<0.001
**Triglycerides**	125.7 (76.0)	93.1 (45.6)	<0.0001
**Glycaemia**	93.4 (21.5)	88.3 (16.0)	<0.0001
**AST**	29.0 (17.5)	18.7 (11.6)	<0.0001
**ALT**	24.4 (13.3)	18.2 (7.9)	<0.0001
**GGT**	32.7 (31.8)	18.8 (16.3)	<0.0001
**Creatinine**	0.86 (0.17)	0.68 (0.14)	<0.0001
	**%**	**%**	***p*** **-value**
**18–29 years**	14.4	19.4	<0.0001
**30–39 years**	26.6	28.9	
**40–49 years**	33.6	32.0	
**50–59 years**	21.5	16.8	
**60–69 years**	3.9	2.9	
**Social class I**	6.1	7.5	<0.0001
**Social class II**	14.5	20.5	
**Social class III**	79.4	72.0	
**Non-smokers**	67.5	66.7	<0.001
**Smokers**	32.5	33.3	
**Obesity I**	15.4	12.7	<0.001
**Obesity II**	3.9	4.6	
**Obesity III**	1.2	1.8	

SBP: systolic blood pressure. DBP: diastolic blood pressure. HDL-c: high-density lipoprotein cholesterol. LDL-c: low-density lipoprotein cholesterol. AST: aspartate aminotransferase. ALT: alanine aminotransferase. GGT: gamma glutamyl transpeptidase.

**Table 2:** Mean values of the different NAFLD and liver fibrosis risk scales according to values of insulin resistance risk scales.

		FLI	HSI	ZJU	FLD	FSI	LAP	BARD
Men	*n*	Mean (SD)	Mean (SD)	Mean (SD)	Mean (SD)	Mean (SD)	Mean (SD)	Mean (SD)
**TG/HDL-c normal**	78,591	28.8 (21.7)	35.4 (6.2)	35.4 (4.9)	30.4 (4.7)	0.1 (0.1)	21.0 (12.3)	0.7 (0.8)
**TG/HDL-c high**	46,812	57.9 (24.5)	39.2 (6.9)	40.1 (5.7)	34.8 (5.3)	0.3 (0.2)	55.0 (35.8)	1.9 (1.0)
**TyG index normal**	90,306	31.1 (22.7)	35.7 (6.3)	35.6 (4.9)	30.7 (4.8)	0.1 (0.1)	22.9 (13.7)	0.8 (0.8)
**TyG index high**	35,097	61.8 (23.5)	39.7 (6.9)	41.0 (5.7)	35.4 (5.4)	0.4 (0.2)	61.3 (38.3)	2.1 (1.0)
**METS-IR normal**	112,656	34.1 (22.8)	35.6 (5.9)	35.9 (4.5)	30.9 (4.3)	0.2 (0.1)	28.6 (22.2)	1.0 (1.0)
**METS-IR high**	12,747	81.5 (14.1)	45.6 (6.3)	46.2 (5.3)	40.6 (5.0)	0.5 (0.2)	71.4 (43.1)	2.4 (0.9)
**Women**	***n***	**Mean (SD)**	**Mean (SD)**	**Mean (SD)**	**Mean (SD)**	**Mean (SD)**	**Mean (SD)**	**Mean (SD)**
**TG/HDL-c normal**	81,396	15.8 (19.1)	35.6 (6.5)	36.1 (5.6)	29.3 (5.4)	0.1 (0.1)	15.4 (11.7)	0.48 (0.67)
**TG/HDL-c high**	12,678	42.7 (27.9)	40.8 (7.6)	42.0 (6.8)	34.9 (6.5)	0.3 (0.2)	44.2 (29.5)	1.65 (0.98)
**TyG index normal**	81,315	15.8 (19.2)	35.6 (6.5)	36.0 (5.5)	29.3 (5.4)	0.1 (0.1)	15.4 (11.8)	0.5 (0.7)
**TyG index high**	12,759	42.7 (27.8)	40.8 (7.7)	42.4 (6.8)	34.9 (6.5)	0.3 (0.2)	44.1 (29.4)	1.7 (1.0)
**METS-IR normal**	87,048	18.6 (21.1)	36.1 (6.6)	36.7 (5.8)	29.8 (5.5)	0.1 (0.1)	18.6 (17.0)	0.6 (0.8)
**METS-IR high**	7026	91.6 (7.9)	55.5 (5.5)	56.2 (5.0)	48.7 (4.7)	0.7 (0.2)	72.5 (34.5)	1.8 (0.9)

TG: triglyceride. HDL-c: high-density lipoprotein cholesterol. TyG: triglyceride–glucose. METS-IR: metabolic score for insulin resistance. FLI: fatty liver index. HSI: hepatic steatosis index. ZJU: Zhejiang University. FLD: fatty liver disease index. FSI: Framingham steatosis index. LAP: lipid accumulation product. In all cases the differences are statistically significant (*p* < 0.0001).

**Table 3:** Prevalence of high values of the different NAFLD and liver fibrosis risk scales according to values of insulin resistance risk scales.

		FLI High	HSI High	ZJU High	FLD High	LAP High	BARD High
Men	*n*	%	%	%	%	%	%
**TG/HDL normal**	78,591	11.1	39.7	24.8	60.2	19.4	16.5
**TG/HDL high**	46,812	49.2	66.0	60.9	63.0	78.2	62.8
**TyG index normal**	90,306	13.5	42.0	26.7	61.3	24.7	18.5
**TyG index high**	35,097	55.7	68.7	68.0	61.2	84.2	73.0
**METS-IR normal**	112,656	17.2	43.9	31.3	7.3	35.1	27.9
**METS-IR high**	12,747	96.9	99.2	100.0	67.4	96.3	85.7
**Women**	***n***	**%**	**%**	**%**	**%**	**%**	**%**
**TG/HDL normal**	81,396	5.3	40.4	30.1	43.1	20.6	8.0
**TG/HDL high**	12,678	29.7	72.3	69.9	53.3	77.2	55.3
**TyG index normal**	81,315	5.3	40.4	29.7	43.1	20.6	7.6
**TyG index high**	12,759	29.2	72.3	72.1	53.4	77.0	57.6
**METS-IR normal**	87,048	2.3	40.3	30.3	1.6	22.7	11.4
**METS-IR high**	7026	85.8	99.9	100.0	48.0	96.2	50.9

TG: triglycerides. HDL-c: high-density lipoprotein cholesterol. TyG: triglyceride–glucose. METS-IR: metabolic score for insulin resistance. FLI: fatty liver index. HSI: hepatic steatosis index. ZJU: Zhejiang University. FLD: fatty liver disease index. FSI: Framingham steatosis index. LAP: lipid accumulation product. In all cases the differences are statistically significant (*p* < 0.0001).

**Table 4:** Binomial logistic regression.

	FLI High	HSI High	ZJU High	FLD High	LAP High	BARD High
	OR (95% CI)	OR (95% CI)	OR (95% CI)	OR (95% CI)	OR (95% CI)	OR (95% CI)
**TG/HDL normal**	1	1	1	1	1	1
**TG/HDL high**	3.95 (3.79–4.11)	1.67 (1.63–1.72)	1.63 (1.58–1.68)	2.37 (2.30–2.45)	5.40 (5.24–5.56)	3.46 (3.35–3.57)
**TyG index normal**	1	1	1	1	1	1
**TyG index high**	2.86 (2.74–2.98)	1.63 (1.57–1.68)	3.07 (2.97–3.18)	1.35 (1.30–1.39)	4.33 (4.18–4.48)	5.07 (4.90–5.23)
**METS-IR normal**	1	1	1	1	1	1
**METS-IR high**	32.35 (31.10–33.61)	18.12 (17.70–18.54)	7.95 (7.78–8.13)	22.13 (21.94–22.32)	42.20 (39.10–45.56)	5.73 (5.51–5.95)

TG: triglycerides. HDL-c: high-density lipoprotein cholesterol. TyG: triglyceride–glucose. METS-IR: metabolic score for insulin resistance. FLI: fatty liver index. HSI: hepatic steatosis index. ZJU: Zhejiang University. FLD: fatty liver disease index. FSI: Framingham steatosis index. LAP: lipid accumulation product. In all cases the differences are statistically significant (*p* < 0.0001).

**Table 5:** Areas under the curve of insulin resistance risk scales to predict the presence of high values of NAFLD and liver fibrosis risk scales.

	FLI High	HSI High	FLD High	LAP High	BARD High
	AUC (95% CI)	AUC (95% CI)	AUC (95% CI)	AUC (95% CI)	AUC (95% CI)
**TG/HDL**	0.832 (0.830–0.835)	0.678 (0.676–0.680)	0.592 (0.590–0.595)	0.874 (0.872–0.875)	0.824 (0.821–0.826)
**TyG index**	0.832 (0.830–0.835)	0.679 (0.677–0.681)	0.593 (0.590–0.595)	0.878 (0.876–0.879)	0.832 (0.830–0.835)
**TyG-BMI**	0.966 (0.965–0.967)	0.901 (0.900–0.902)	0.673 (0.670–0.676)	0.927 (0.926–0.928)	0.880 (0.878–0.882)
**TyG-waist**	0.969 (0.968–0.970)	0.793 (0.791–0.795)	0.615 (0.612–0.617)	0.943 (0.942–0.944)	0.840 (0.838–0.842)
**TyG-WtHR**	0.972 (0.971–0.972)	0.829 (0.827–0.831)	0.619 (0.616–0.621)	0.962 (0.961–0.963)	0.858 (0.856–0.859)
**METS-IR**	0.957 (0.956–0.957)	0.897 (0.896–0.899)	0.669 (0.667–0.672)	0.905 (0.903–0.906)	0.871 (0.869–0.873)

TG: triglycerides. HDL-c: high-density lipoprotein cholesterol. TyG: triglyceride–glucose. METS-IR metabolic score for insulin resistance. BMI: body mass index. WtHR: waist-to-height ratio. FLI: fatty liver index. HSI: hepatic steatosis index. ZJU: Zhejiang University. FLD: fatty liver disease index. FSI: Framingham steatosis index. LAP: lipid accumulation product. AUC: areas under the curve.

## Data Availability

Not applicable.
